# The Law of the Similars

**Published:** 1883-12

**Authors:** 


### BOOK NOTICES.

THE LAW OF SIMILARS, ITS DOSAGE AND THE ACTION OF ATTENUATED MEDICINES. By C. Wesselhœft, M. D. Otis Clapp & Sons. 1883.

This little book gives in a very interesting manner a condensation of several lectures delivered before the Boston University School of Medicine. The most peculiar features of the lectures are Dr. Wesselhœft's extreme views as to potency. On this point he dogmatizes with a *vim*. Once upon a time, not so long ago either, Dr. W. reported cases cured by the high potencies. Were they myths? Dr. W. does not give correctly Hahnemann's views on the repetition of the dose.

